# Supplementary figures and images for: Cell wall dynamics stabilize tip growth in a filamentous fungus
Source: PLoS Biol. 2023 Jan 17;21(1):e3001981. doi: 10.1371/journal.pbio.3001981 (PMC9882835; doi:10.1371/journal.pbio.3001981)

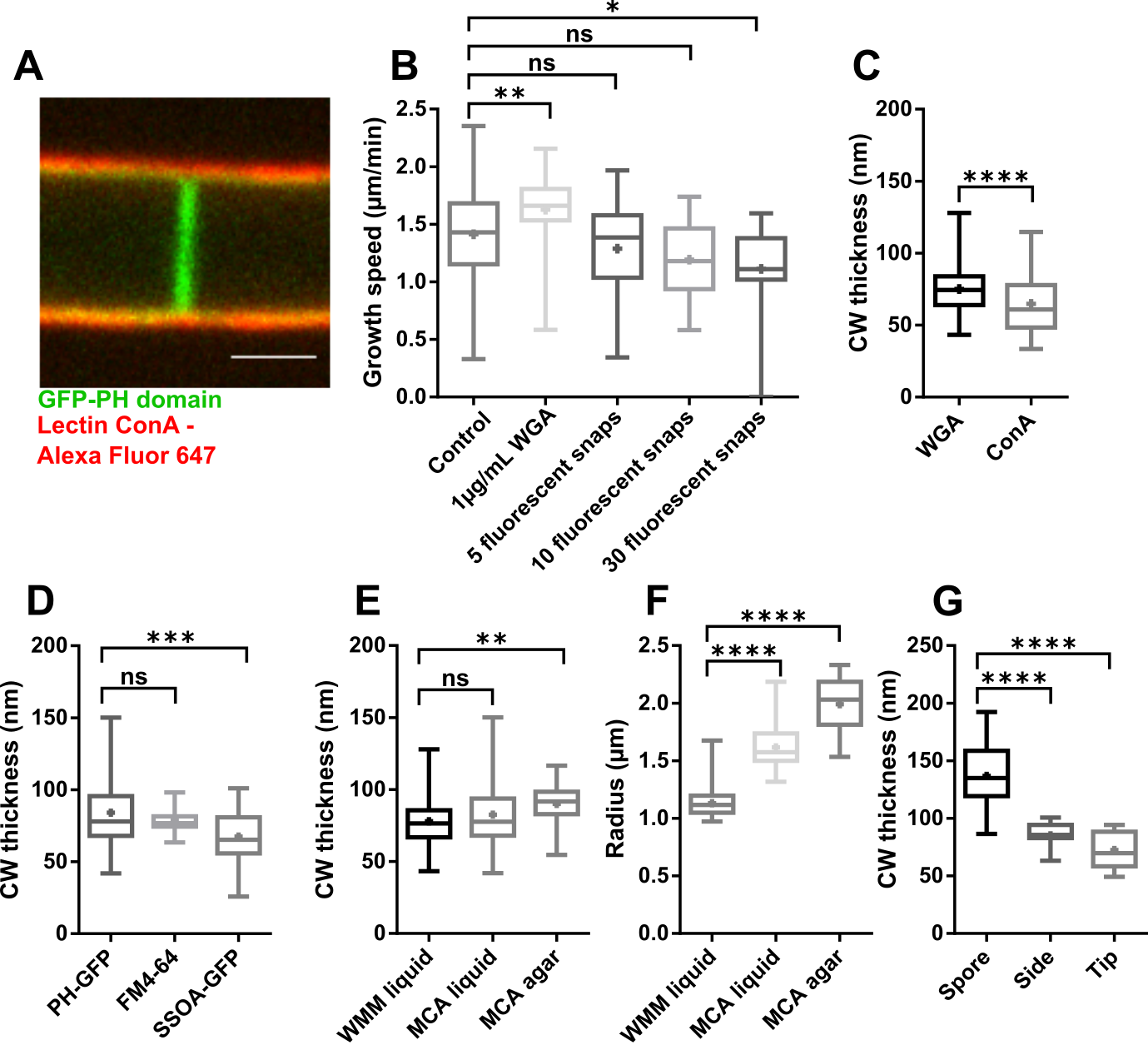

Supplement: S1 Fig — (A) Mid-slice confocal image of a dividing hyphae, stained with fluorescent lectins, demonstrating that lectins only decorate exposed polysaccharides at the cell surface, and not internal CWs at the septum. (B) Impact of lectin (WGA) and fluorescent imaging on cell growth speeds (n > 15 cells in each condition). (C) CW thickness measured with 2 different lectins: WGA (n = 82 cells) or ConA (n = 89). (D) CW thickness measurement using PH-GFP to label the plasma membrane (n = 103) or other plasma membrane marker: the FM4-64 dye (n = 25) or a strain expressing the membrane-associated synaptobrevin protein SSOA-GFP (n = 49). (E) CW thickness of cells grown in minimal liquid media (WMM, n = 53), rich liquid media (MCA, n = 81), or rich solid media (MCA, n = 19), using the WGA lectin. (F) Cell radii in the same conditions as in E. (G) CW thickness of spores, cells sides, and tips in germling tubes (n = 24, 11 and 11 cells). Scale bar, 2 μm. Error bars correspond to +/− SD. Results were compared by using a two-tailed Mann–Whitney test. n.s, P > 0.05; **, P < 0.01, ***, P < 0.001, ****, P < 0.0001. The data underlying the graphs can be found in S1 Data. ConA, Concanavalin A; CW, cell wall; WGA, wheat germ agglutinin; WMM, watch minimal medium. (TIF) [file pbio.3001981.s001.tif]

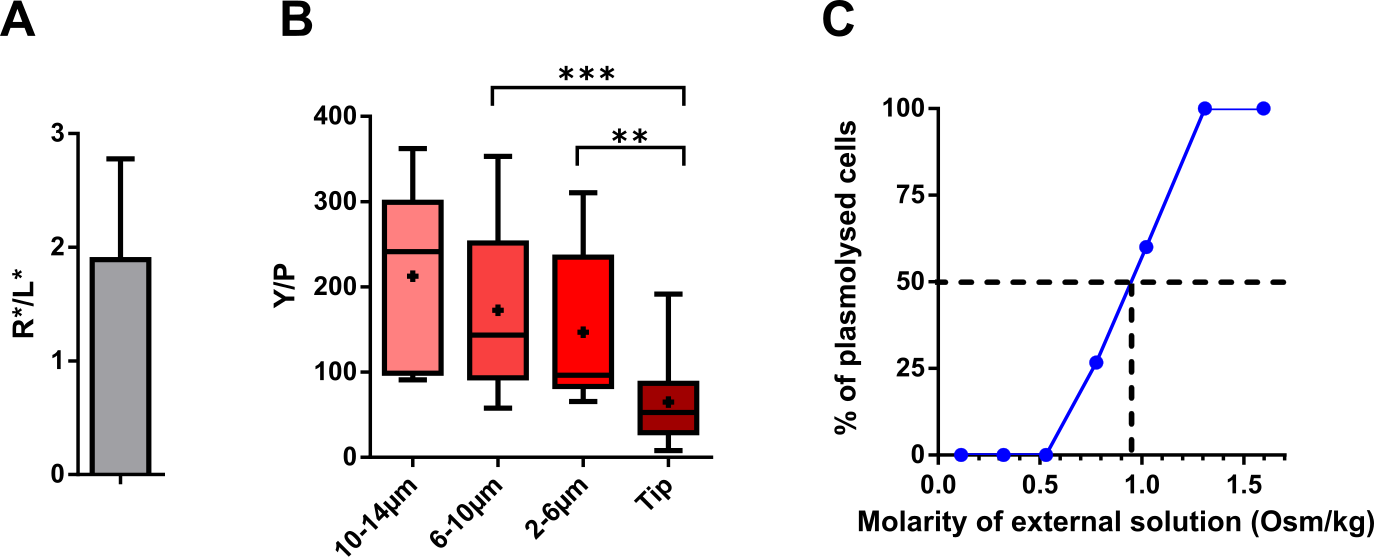

Supplement: S2 Fig — (A) Ratio between the radius and the longitudinal shrinkage (elastic strain) during photoablation assay (n = 10 cells). A ratio close to 2 suggests the abscence of major anisotropies in the CW. (B) CW Young’s divided by turgor pressure, measured along mature hyphae (n = 7 at least for each compartments). (C) Turgor pressure measurement using the percentage of plasmolyzed cells (in which the plasma membrane detaches from the CW) as a function of medium osmolarity (n = 17 cells at least for each molarity). Error bars correspond to +/− SD. Results were compared by using a two-tailed Mann–Whitney test. **, P < 0.01, ****, P < 0.0001. The data underlying the graphs can be found in S1 Data. CW, cell wall. (TIF) [file pbio.3001981.s002.tif]

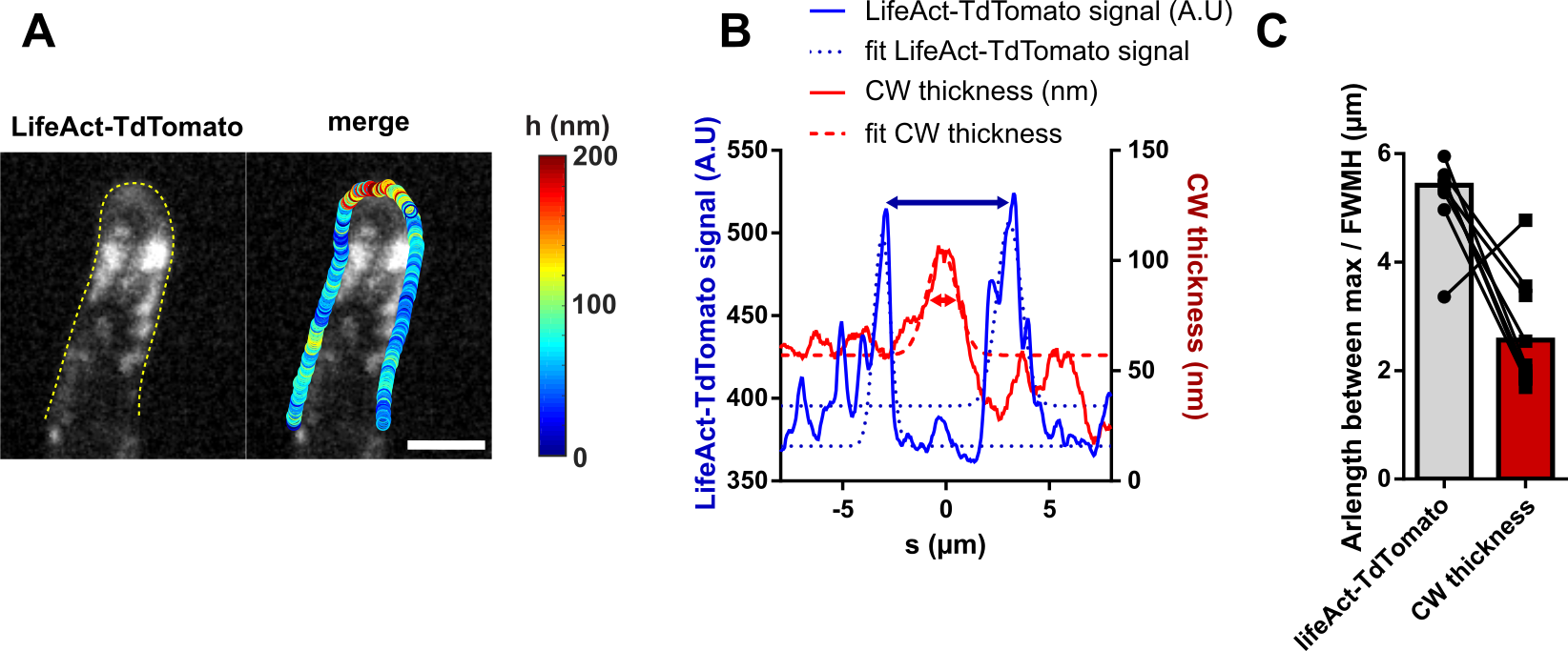

Supplement: S3 Fig — (A) Maximum projection of cells expressing LifeAct-TdTomato overlaid with the CW thickness profile. (B) Measurement of the arclength distance between the two maxima corresponding to the endocytic collar, and the FWMH of the CW thickness at cell tip. (C) Comparison between the distance between the two maxima of LifeAct signal and the FWMH of the CW thickness. Each black line corresponds to a single cell. The data underlying the graphs can be found in S1 Data. CW, cell wall; FWMH, full width at mid height. (TIF) [file pbio.3001981.s003.tif]

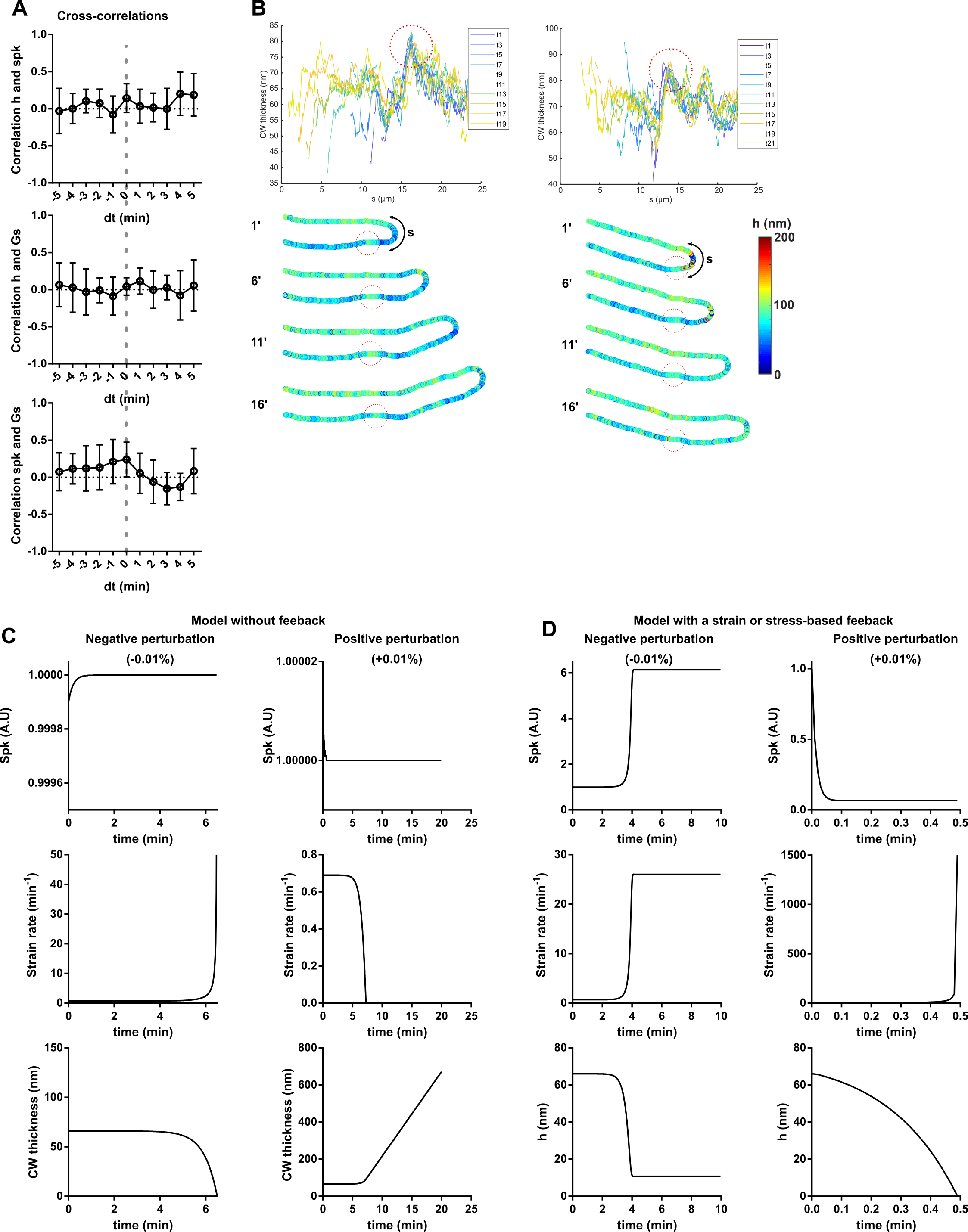

Supplement: S4 Fig — (A) Temporal cross-correlation, as a function of different time delays between pairs of the three parameters: EVs concentration, the strain rate, and the tip CW thickness (n = 10 time lapses). Postive values stand for correlations; negative values are anticorrelations; and null values suggest the absence of any correlationat timescales assayed. Cross-correlations are computed as <(h(t)−)(EV(t+dt)−)>σhσEV. (B) Two representatives examples of tracking of a fiducial thickness mark where the CW is locally thicker (indicated by dotted circles) during hyphal growth, and corresponding thickness profiles plotted a function of s for different time points in the movie (color coded) and shifted to substract cell growth. The alignment of the peaks suggests that the mark is fixed with respect to the lab referential and, thus, the absence of major CW advective backward flows. (C) Simulation of the effect of a small postive or negative perturbation of the EV level on strain rate and CW thickness dynamics in the absence of mechanical feedback in the model. (D) Simulation of the effect of a small postive or negative perturbation of the EV level on strain rate and CW thickness dynamics in a model with a strain/stress-based feedback instead of a strain rate–based feedback. Scale bar, 2 μm. Error bars correspond to +/− SD. The data underlying the graphs can be found in S1 Data. CW, cell wall; EV, exocytic vesicle. (TIF) [file pbio.3001981.s004.tif]

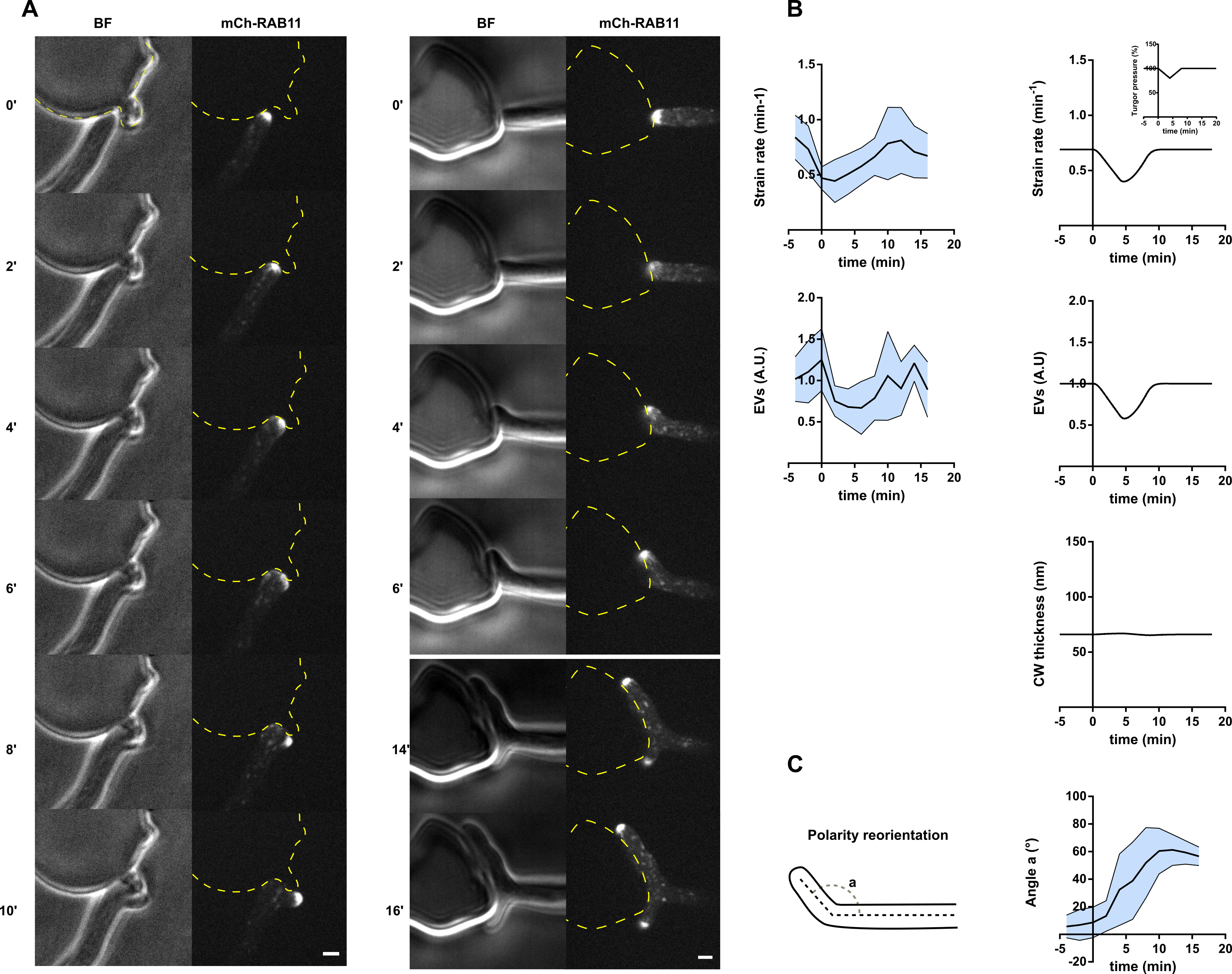

Supplement: S5 Fig — (A) Examples of hyphae growing in PDMS microchannels that contact microfabricated posts, delineated in the fluorescence channel with yellow dotted lines. Note how EVs labeled with mCherry-RAB11 exhibit a partial dispersal followed by a domain reformation away from the site of contact. (B) Average dynamic evolution CW strain rate and mCherry-RAB11 levels (EVs concentration) (n = 6) and corresponding model outputs. The origin of time is defined as the time of contact. The CW thickness is not measured experimentally due to technical limitation in microchamber. (C) Dynamic evolution of the angle of polarity reorientation, computed as the angle with respect to the initial growth axis (n = 6). Scale bars, 2 μm. The data underlying the graphs can be found in S1 Data. CW, cell wall; EV, exocytic vesicle; PDMS, polydimethylsiloxane; (TIF) [file pbio.3001981.s005.tif]

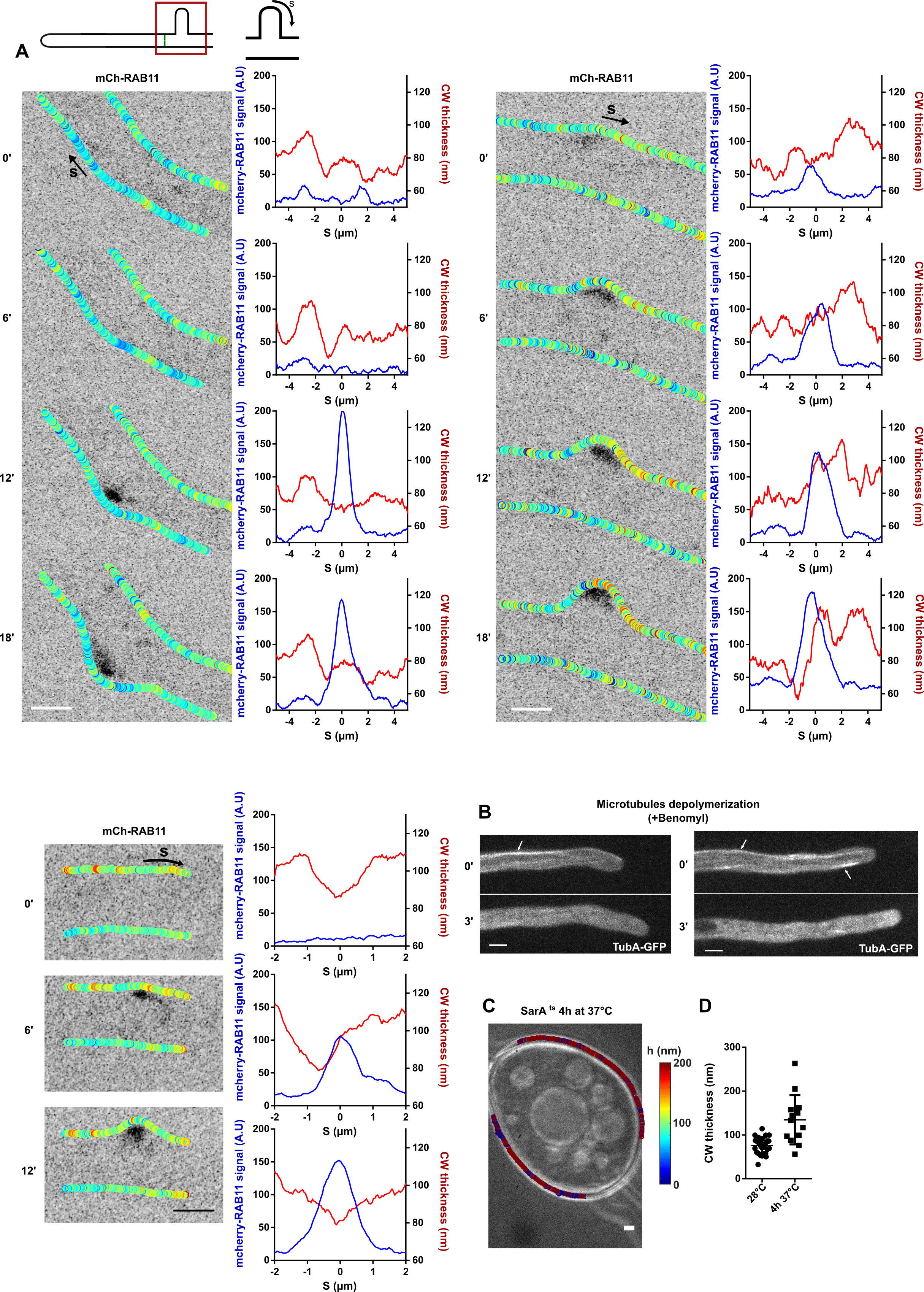

Supplement: S6 Fig — (A) Examples of branching events. Left: CW thickness map overlaid on the EVs (mCherry-RAB11) signal. Right: Intensity profile of the EVs and CW thickness along the cell side, with s = 0 corresponding to the incipient branching site. (B) Depolymerization of microtubules (marked with white arrows) visualized using a strain expressing TubA-GFP upon benomyl treatment. (C) Brigth field image overlaid with CW thickness maps of a sarA6 ts cell grown 4 h at 37°C. (D) Mean CW thickness of sarA6 ts grown at permissive temperature (28°C) or restrictive temperature (37°C) for 4 h. Scale bar, 2 μm. Error bars correspond to +/− SD. The data underlying the graphs can be found in S1 Data. CW, cell wall; EV, exocytic vesicle. (TIF) [file pbio.3001981.s006.tif]

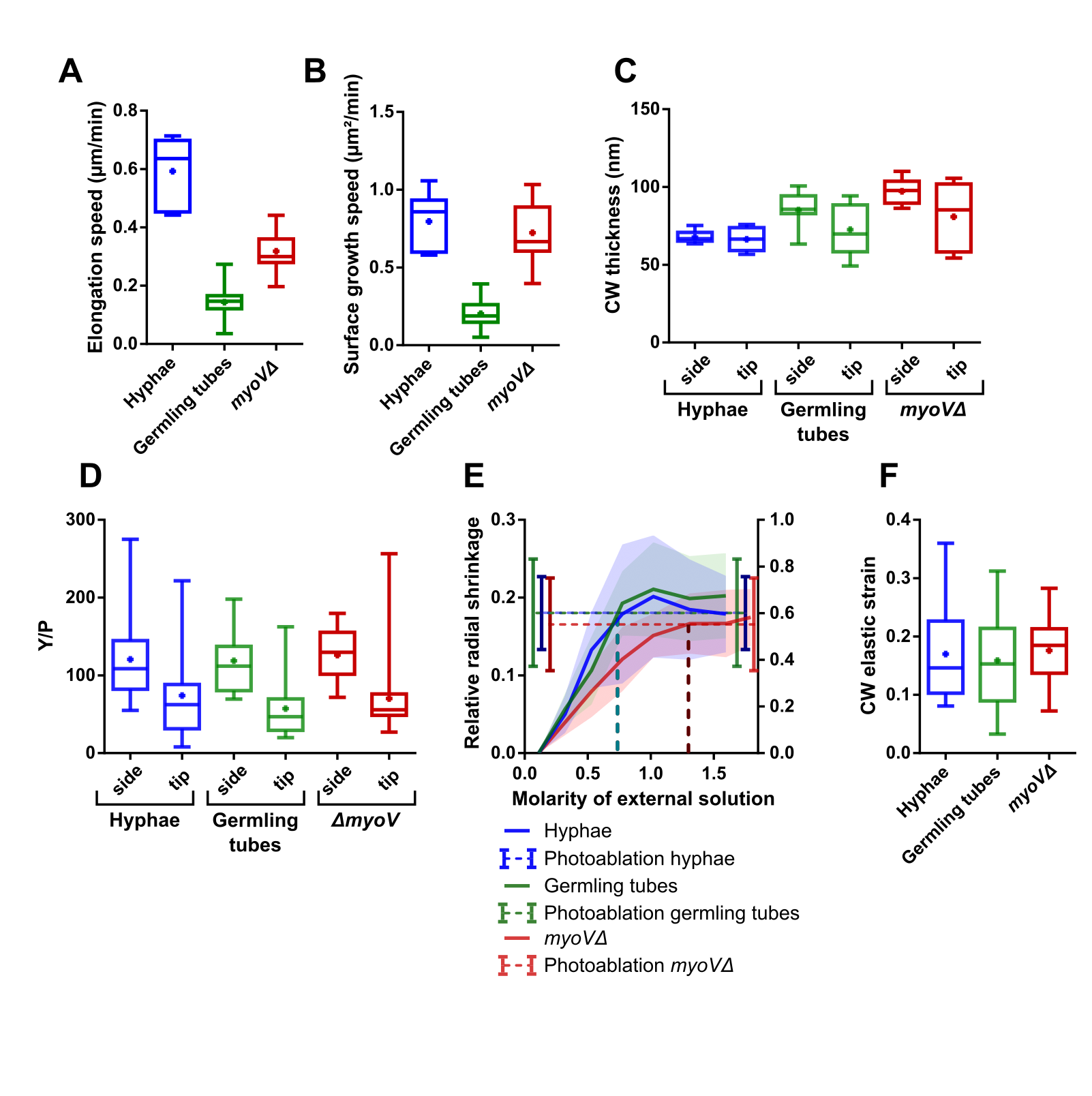

Supplement: S7 Fig — (A) Linear elongation speed of mature hyphae, germling tubes, and myoVΔ cells. (B) Surface growth speed of mature hyphae, germling tubes, and myoVΔ cells. (C-D) CW thickness (C) and CW Young’s modulus divided by turgor pressure (Y/P) (D), of mature hyphae, germling tubes, and myoVΔ cells, at cell tips vs. cell sides. (E) Turgor pressure measurement from CW lateral elastic strains as a function of medium osmolarity of mature hyphae, germling tubes, and myoVΔ cells. (F) Tip CW elastic strains of mature hyphae, germling tubes, and myoVΔ cells. The data underlying the graphs can be found in S1 Data. CW, cell wall. (TIF) [file pbio.3001981.s007.tif]
