# Supplementary material for: Cell wall dynamics stabilize tip growth in a filamentous fungus
Source: PLoS Biol. 2023 Jan 17;21(1):e3001981. doi: 10.1371/journal.pbio.3001981 (PMC9882835; doi:10.1371/journal.pbio.3001981)
Supplement: S1 Table — (DOCX) [file pbio.3001981.s008.docx]

## **Table S1**. *Aspergillus nidulans* strains used in this study

| Genotype | Number |
| --- | --- |
|  |  |
| *pabaA*1 *yA*2; *argB*2[*argB**-*gpdA*^p^::*gfp*::PH-*PLC*dx2] | MAD2471 |
| *pabaA*1; *nkuA*∆::*bar* | MAD6525 |
| *pyrG*89?; *argB2*::[*argB**-*gpdA*^p^::*gfp*::2xPHdomain of *PLCd*1]; Δ*nkuA*::*bar*?; *rab11*^P^::*mCherry*::*rab11*::*pyrG^Af^*::3´UTR*rab11* | MAD7306 |
| *pyrG*89?; *sarA*6; *argB2*::[*argB**-*gpdA*^p^::*gfp*::2xPHdomain of *PLCd*1] Δ*nkuA*::*bar*?; *rab11*^P^::*mCherry*::*rab11*::*pyrG^Af^*::3´UTR*rab11* | MAD7476 |
| *pyrG*89?; Δ*myoE*::*pyrG^Af^* *argB2*::[*argB**-*gpdA*^p^::*gfp*::2xPHdomain of *PLCd*1]; *pyroA*4 Δ*nkuA*::*bar*?; *rab11*^P^::mCherry::*rab11*::*pyrG^Af^*::3´UTR*rab11* | MAD 7480 |
| *pyrG*89?; *argB*2::[*argB**-*gpdA*p-*gfp*-2xPHdomain of *PLCd*1]; *pyroA*4 Δ*nkuA*::*bar*?; *pyrG^Af^*::*mCherry*::*chsB* | MAD7473 |
| *pyrG*89?; *myoE*-*mCherry*::*pyrG^Af^* *argB2*::[*argB**-*gpdA*p-*gfp*-2xPHdomain of *PLCd*1]; Δ*nkuA*::*bar*? | MAD7562 |
| *pyrG*89, *wA*::*gfp*::t*ubA*::*pyrG^Af^*, *pyroA*4 *nkuA*∆::*argB*2 | MAD4658 |
| *pabaA*1; *inuA*^P^::lifeact::*tdtomato*::*riboB^Af^*::*inuA*^T^; *argB*2::[*argB**-*gpdA*p-*gfp*-2xPHdomain of *PLCd*1]; *nkuA*Δ::*bar*?; *riboB*2? | MAD7474 |
