# Supplementary material for: Cell wall dynamics stabilize tip growth in a filamentous fungus
Source: PLoS Biol. 2023 Jan 17;21(1):e3001981. doi: 10.1371/journal.pbio.3001981 (PMC9882835; doi:10.1371/journal.pbio.3001981)
Supplement: S2 Table — (DOCX) [file pbio.3001981.s009.docx]

**Table S2**: Model parameters

|  | Mature Hyphae | Benomyl | *sarA6* | Pressure drop and obstacle | Germling  tubes | *myoVΔ* |
| --- | --- | --- | --- | --- | --- | --- |
|  |  |  |  |  |  |  |
| γ | 0.04554 | 0.04554 | 0.04554 | 0.04554 | 0.078214 | 0.066913 |
| η | 0.014811 | 0.014811 | 0.014811 | 0.014811 | 0.002801 | 0.003647 |
| α | 9.30581 | 1.768104 | 0.790994 | 9.30581 | 1.18309 | 1.5781 |
| ϕ | 13.4867 | 2.69734 | 1.213803 | 13.4867 | 1.10422 | 1.91033 |
| θ | 13.03 | 13.03 | 13.03 | 13.03 | 9.51 | 8.86 |
