# Supplementary material for: Cell wall dynamics stabilize tip growth in a filamentous fungus
Source: PLoS Biol. 2023 Jan 17;21(1):e3001981. doi: 10.1371/journal.pbio.3001981 (PMC9882835; doi:10.1371/journal.pbio.3001981)
Supplement: Attachment — Submitted filename: Reviews_Rebutal_MINC.pdf [file pbio.3001981.s020.pdf]

Nicolas Minc, PhD
CNRS, Research Director
Group Leader “Cellular Spatial Organization”
Institut Jacques Monod, UMR7592
15, rue Hélène Brion
75205 PARIS CEDEX 13
Tel : 33 1 57278052
nicolas.minc@ijm.fr

November, 24th 2022

Dear Editors at *Plos Biology*,

We are pleased to enclose a revised version of our manuscript **“Cell Wall Dynamics in a Filamentous Fungus”** for your consideration as a research article in *Plos Biology*. We have addressed all the reviewers’ comments with new figures and text edits.

The main changes in the MS include:

1- As asked by Reviewer #3, we have now provided further evidence that cell wall (CW) mechanics and mechanical deformations are essential elements in the regulation of CW secretion and synthesis. For this, we developed microfluidic obstacles to directly hinder tip growth mechanically. As presented in novel Figure S5 and Movie S3, we found that hyphal cells slow down growth when growing against this physical barrier which causes the rapid detachment of secretory vesicles, and their subsequent reassembly along an angle nearly orthogonal to the obstacle to promote CW assembly and tip growth in a mechanically favorable direction.

2- To address another concern of Reviewer #3 on the plausible nature of mechanical feedbacks, we have now tested models based on feedbacks from CW elastic strains or stress. As shown in new Figure S4, this class of model generally leads to explosive behavior with rapidly increasing growth rates and vanishing CW thickness. These results support that a general feedback from CW strain rates onto vesicle accumulation may represent the simplest hypothesis to stabilize hyphal tip growth as well as promote morphogenetic plasticity.

3- We have addressed remaining points with text edits which are highlighted in red in the revised manuscript.

We thank the editors and reviewers for their valuable inputs and comments that have greatly helped us to improve this manuscript.

We thank you for your consideration,

Sincerely yours,

Nicolas Minc

Reviewers' comments

Rev. 1:
This article, by Dr. Minc, Boudaoud, and coworkers, presents a beautiful study of cell wall dynamics. The method and vision of this study is very unique, from a physical/biophysical perspective. The manuscript mapped out the cell wall thickness, stiffness of the hyphae of living A. nidulans, which provides insight into the mechanical properties associated with hyphal tip growth. It is then followed by a discussion of the cell wall secretion by monitoring the vesicle accumulation.

Though from a physical view, the manuscript is well written and should be fully accessible to any reader of PLOS Biology. I enjoyed reading the manuscript, especially for the well-organized discussions where the methodology merit, the link of secretory vesicles and cell wall balance, and cell wall mechanical feedbacks, are discussed in great details.
The figures are highly attractive and can efficiently support the major statements of this manuscript. Considering all the merits, I suggestion publication on PLOS Biology. I believe it will be an important addition to the literature and will attract readers from many research directions, in particular, cell wall structural biology and microbiology.

We thank the reviewer for her/his positive assessment of our work and for endorsing publication of the manuscript.

There is a minor question regarding the section of "A spatial gradient of cell wall stiffness is associated to hyphal polar growth," the authors mentioned that CW thickness polarity could be inverted in a time-course as short as ~few min. It will be very helpful if the authors could clarify here what is the time resolution regarding the method.

We have now expanded the description of these results by directly specifying the time-resolution used in the time-lapses considered for these observations.

Rev. 2:
Fungal tip growth is a subject of considerable importance. Fungi are very serious human, veterinary and plant pathogens, killing roughly 1.5 million persons each year and about 10% of harvested crops are spoiled by fungi. On the other hand fungi produce billions of dollars of industrial enzymes, small molecules such as citric acid and secondary metabolite drugs such as the statins. A great deal of progress in understanding, at a molecular level, how fungi grow has been made in recent years. We know the identities many of the molecules that are required for tip growth and we have a rough outline of the mechanisms of vesicular trafficking, but many key questions remain including the question of how vesicular trafficking is regulated.

The authors employed a method of measuring the thickness of the cell wall based on a method developed in Schizosaccharomyces pombe. It uses fluorescently tagged lectins (Concanavalin A and Wheat Germ Agglutinin) to label the outside of the cell wall and GFP-tagged Pleckstrin Homology domain to label the plasma membrane. The authors used spinning disk confocal microscopy with gaussian fitting to determine wall thickness with resolution below that of the traditional (Abbé) limit of resolution. Essentially, the distance between the gaussian peaks of the GFP-Pleckstrin Homology domain and the lectin is taken as

the wall thickness. The values obtained are similar to those obtained with electron microscopy after chemical fixation or rapid freezing and the technique appears to give valid data. The technique is powerful because it allows one to measure wall thickness in living material and, in the same cells at the same time, look at dynamics of important components of the tip growth apparatus such as secretory vesicles.
Perhaps surprisingly, thickness of the cell wall did not differ a great deal between the hyphal tip and the hyphal wall. Thickness did not change very much as a function of speed, and although tip cell wall thickness was generally slightly less than the thickness of the hyphal wall, this was not always the case, which suggests that cell wall thickness gradients do not establish polarity in tip growth. By perturbing tip growth in a number of ways (depolymerizing microtubules with benomyl, using a MyoV deletant, laser ablation, altering osmolarity), the authors were able to calculate or estimate a number of values (e.g. Young's modulus), draw a number of conclusions, and develop a mathematical model for tip growth.
There is no point in reiterating all of the data and conclusions in the manuscript, but some of the main conclusions are as follows.
The position of secretory vesicles is a good indicator of cell wall remodeling and speed of tip growth, but turgor and cell wall mechanics do not correlate well with tip growth speed. Relatedly, there is a balance of secretory vesicle recruitment and tip expansion.
During lateral branch growth, there appears to be a positive feedback mechanism that leads to an accumulation of secretory vesicles.
There is a spatial gradient of cell wall stiffness with cell tip walls being less stiff than lateral walls (presumably reflecting cross-linking of cell wall components?).
The model requires a mechanical feedback loop and a number of findings indicate a positive feedback mechanism that coordinates tip growth with vesicle trafficking. For example, impairing vesicle trafficking does not result in a thinner wall, as one might intuitively assume, but, rather a thicker wall (i.e. tip growth is slowed to a greater extent than vesicle trafficking). There are plausible mechanisms that might employ conserved fungal mechanosensors.

This manuscript combines biology, sophisticated microscopy and mathematical modeling to give fresh insights into an important phenomenon. I feel that I am quite competent to evaluate the microscopy and biology, and I believe they are sound and worth of publication in a high profile journal. While the mathematical model leads to biologically plausible conclusions, I don't feel that I am capable of reviewing the mathematics in this manuscript. Indeed, one problem with multidisciplinary approaches is that it is difficult for individuals to evaluate them. Teams are really necessary. Assuming that other reviewers are happy with the mathematics, I am happy to recommend publication in PLoS Biology.

We thank the reviewer for her/his positive assessment of our work and for endorsing publication. Following reviewer’s comments, we have now added a sentence to propose that the higher stiffness of lateral cell walls may indeed plausibly reflect a different cross-linking of cell wall components.

Rev. 3:
This work studies the dynamics of cell wall secretion, remodeling, and deformation of fungus hyphae. The authors developed a live-imaging technique to monitor the dynamics of hyphal cell wall (CW) thickness in Aspergillus nidulans by labeling the plasma membrane and the polysaccharides outside the CW. Combining measurement of CW thickness, turgor pressure, and the shape changes after photoablating the CW with a linear elastic model uncovered a small gradient of CW thickness and a larger gradient of CW stiffness from the hyphal tip to the side, with the tip on average being ~13% thinner but ~1/3 of Young's

Modulus compared to the side. Fluorescent labeling of exocytic vesicles showed a similar spatial distribution compared to the CW thickness profile near the tip. Time-lapse imaging revealed low temporal fluctuation (~10-15%) in growth speed, CW thickness and vesicle amount near the tip. Based on these observations, the authors developed a model (ODEs) that uses posited mechanical feedback to connect growth, CW thickness, CW remodeling, and secretory vesicle production to account for steady state hyphal growth. Simulation also agreed semi-quantitatively with four test cases of abrupt changes in growth or secretion (lateral branching, reduced turgor pressure, and altered vesicle trafficking). The authors also fitted the model to the steady state growth of early germling tubes and the mature hyphae of a myoV deletion mutant and found a substantial difference in the coefficient of mechanical feedback.

We thank the reviewer for her/his detailed assessment of our work. We have followed suggestions and addressed all points raised.

Major concerns

1. The experimental data are not enough to back up the mechanistic model that integrates growth, CW thickness, vesicles production, and mechanical feedback. Live-imaging shows a relatively stable growth speed, CW thickness and vesicle amount during steady state growth; mechanical modeling suggests lower stiffness at the tip versus the side. However, the paper does not provide direct evidence of mechanics playing a role in regulating the cell wall synthesis process.

We agree with the reviewer. To further substantiate the role of mechanics and mechanical feedbacks in the regulation of CW secretion, we have developed a novel assay in which hyphal cells are grown against microfabricated obstacles. This provides a more direct mean to mechanically hinder tip growth rates (as a complement of previously presented turgor loss assays).

As presented in novel Figure S5 and Movie S3 we find that when hyphal tips grow against a physical barrier, tip growth slows down, which systematically leads to the transient detachment of secretory vesicles from cell tips. This is always followed by the reformation of a novel front of vesicles away from the obstacle that stabilizes tip growth along a new direction. In addition, in these situations, we often observe apical tip branching (see examples in figure S5A and movie S3), a process which we never observed in normal hyphal tips of *Aspergillus nidulans* growing in free conditions. All these observations strongly suggest that when mechanical barriers oppose tip growth to slow down elongation rates, they dramatically affect secretory vesicle polar accumulation. We note however, that in spite of extensive efforts to map CW thickness in these assays, the presence of the microfabricated obstacles limited lectin diffusion at the contact site, prevented us to compute variations in CW mechanics when tips grow against the physical barrier, and to compare this experimental output with models' predictions.

We hope the reviewer will conquer that these new lines of evidence better support our general claim that mechanics at cell tips is directly relevant to CW secretion and consequent synthesis.

There is no data backing up the mechanical feedback proposed in the model; it is also questionable that the mechanical feedback is based on strain rate-dependent vesicle production/transport, as suggested by the model.

We agree and have now tested other plausible sources of mechanical feedbacks based on putative direct "CW strain or stress sensing". As presented in novel figure S4D, the model with such hypothesis has similar explosive behavior as a model without feedbacks, and generally fails to predict stable hyphal growth observed in experiment. These analyzes suggest that a feedback from strain rates (growth) on secretory

vesicle amount, may provide the simplest assumption to understand both steady hyphal growth as well as rapid morphogenetic adaptation to modulations in growth or secretion.

2. The authors need to clarify the contribution of the work. Experimentally, they developed a live-imaging technique to track cell wall thickness dynamics during growth, measured the turgor pressure, and by mechanics calculation, showed a gradient in stiffness along the hypha. The results are mostly phenomenological. The mechanistic aspect of this work lies mostly in the model and fitting the model to test cases. However, the model itself does not explain its superiority to the existing models. The authors need to elaborate on how the model promotes quantitative understanding of the cell wall synthesis and regulation.

The reviewer questions the key contribution of this work. First, we would like to advocate that, as outlined by the two other reviewers, the experiments and quantitative CW measurements developed here, although admittedly partly phenomenological, are completely novel and unique with respect to the state of the art in fungal biology. As such, the goal of this work is not to discover new molecules or pathways implicated in CW synthesis, but rather provide novel insights into how biochemical activities ultimately shape and regulate CW secretion, mechanics and deformation during hyphal growth.

The model in that regard, is indeed built on the standard resolution of coupled ODEs, as several models in related literature. Yet, to the best of our knowledge, this is the first model that considers and predicts the dynamic evolution of CW thickness, and explores multiple dynamic modulation of secretion, growth or turgor, that can be compared with experimental outputs. In addition, we have inferred model parameters from experimental data and showed that the model can semi-quantitatively reproduce experimental data. Therefore, the novelty is to have developed a model that can essentially predict the most relevant CW dynamic modulations found in fungal cells.

To address this point, we have now added a new paragraph in the discussion to clarify the advance of the model, and also improved other parts of the discussion to better highlight the key contribution of our work with respect to the state of the art.

Minor concerns

1. The authors need to clarify the conclusions of the section "Spatial patterns of secretory vesicle accumulation and cell wall mechanics", which says "CW remodeling at cell tips may be best represented by the global distribution of RAB11 EVs" and "these analyses directly highlight in living cells the spatial relationships between exocytic carrier distribution at cell tips and local modulations in CW synthesis and mechanics". What does it mean and why is it important to "represent" CW remodeling? And why is the "spatial relationship" important?

CW remodeling remains an ill-defined parameter for tip growth, as it may potentially reflect synthesis, mechanical deformation, lytic activities, or other biochemical activities that *e.g.* alter the cross-links between polysaccharides. Therefore, we use CW thickness spatial variations at cell tips as a proxy for CW mechanical changes, and map their spatial extent to that of different canonical proteins that regulate synthesis (ChsB, a chitin synthase), transport (MyoV), endocytosis (Actin patches) and secretory vesicles production (Rab11). A surprising result is to find that these markers exhibit markedly different spatial extent, with the transport system being typically more focused, while synthesis being in contrary broader than CW thickness spatial variations. We have now rephrased this section to better explain the rationale of this approach, the importance and limits of the conclusion drawn. We hope it is clearer.

Is the localization of the exocytic vesicles part of the paper's discovery? Is purpose of this section mainly to justify the involvement of exocytic vesicles in the model to be preposed later?

We agree that this is not a discovery of the paper, and note that that we had clearly stated that this was previously reported in the literature in the original text. We have now better explained that the novelty is to map their spatial extent with that of CW thickness, which indeed justifies to use this signal as a proxy for CW synthesis and remodeling in both experiments and models.

2. In Fig. 4C, the relative standard deviation of the thickness (h) and the EV amount are above the 10-15% the authors claimed in the text.

We thank the reviewer for pointing this out, we have now changed it to “10-20%”.

3. In the section "Dynamic co-evolution of CW thickness, tip growth and exocytosis during hyphal tip shape changes", it says "These patches then fluctuated in intensity and position, reflecting negative feedbacks". Why does this imply negative feedbacks?

The notion of negative feedback in polarity regulation has been established and discussed by many groups especially in the context of Cdc42 polarity establishment in yeast. The community uses this description when polar patches oscillate or flicker or shrink in size which indicate a reversal of patch growth by a negative control (see Ozbudak et al. Dev Cell, 2005, Das el at. Science 2012, or Howell et al. Cell 2012 as examples). As we understand that this may lead to confusion, and is somewhat tangential, we chose to remove this expression.

4. In the simulation of the 2 cases where vesicle accumulation is affected (Fig. 5FGHI), both EV production and degradation need to be reduced (and the degradation coefficient has to drop more than the production coefficient, as indicated in the Supplementary information) in the model to reproduce cell wall thickening. Why would microtubule depolarization/Golgi disassembly affect EV degradation more than production?

We indeed find that the transfer constant α which represents the decay of vesicles by feeding the CW needs to be reduced more than the production term to account for microtubule depolarization/Golgi disassembly experimental results. A plausible interpretation is that these inhibitions also strongly alter the trafficking of exocyst or SNARE components thereby limiting the tethering and/or fusion of vesicles with the plasma membrane and therefore material transfer to the CW.
